# Two-year hospital records of burns from a referral center in Western Iran: March 2010-March 2012 

**DOI:** 10.5249/jivr.v6i1.276

**Published:** 2014-01

**Authors:** Touraj Ahmadijouybari, Farid Najafi, Mehdi Moradinazar, Behzad Karami-matin, Reza Karami-matin, Maria Ataie, Masoumeh Hatami, Samira Purghorbani, Vahid Amee

**Affiliations:** ^*a*^Imam Khomaini Hospital, Kermanshah University of Medical Sciences, Kermanshah, Iran.; ^*b*^School of Public Health, Kermanshah University of Medical Sciences, Kermanshah, Iran.

**Keywords:** Unintentional burns, Self-immolation, Epidemiology, Kermanshah, Trends

## Abstract

**Background:**

Burns are among the most common injuries affecting a great number of people worldwide annually. In Iran, especially in its western region and in Kermanshah province, burns have a relatively high incidence. The present study was aimed at investigating epidemiological characteristics in Western Iran.

**Methods:**

Within a cross-sectional study, the data on all patients attending the Burns Center at Imam Khomeini Hospital (Kermanshah, Iran) during 2010-2011 and 2011-2012 (24 months) were collected. Then, age, gender, cause of burns, total body surface area, and time of the occurrence were extracted from the hospital records. The data were analyzed using the SPSS statistical package (Version 19, for Windows). We used chi-squared test when we compared the categorical responses between two or more groups. For comparing means between two groups we used t-test. In addition, trends were investigated using linear regression.

**Results:**

Overall 13 248 people were referred to the Burns Center at Imam Khomeini Hospital (Kermanshah, Iran) during the period of study, including 328 cases of self-immolation. The mean age of the patients was 27±19 years and 29±13 years for unintentional burns and self-immolation respectively. Out of the total number of unintentional cases, 6 519 (50.5%) were men, while the corresponding percentage of men among the self-immolation cases was 16.6% (p less than 0.001). Trends in the number of cases were cyclic, with the highest and lowest number of burns cases being in March and May. Overall, hot liquids and flammable materials were the two most important causes of unintentional burns. However, flammable materials were the main cause of burns among self-immolation cases. During hospital admission, 168 (51%) self-immolation victims and 43 (0.33%) unintentional burn victims died.

**Conclusions:**

While major preventive measures are not adequately used in developing countries, burns and their burden can be significantly reduced by increasing public awareness and by applying simple preventive measures.

## Introduction

Burns are among the most common injuries affecting a great number of people of different ages and cultures every year. According to World Health Organization statistics, 5-12% of the total injuries in the world are related to burn.^1,2^ It is estimated that 2.5 million Americans suffer from burn injuries annually.^3-5^ Studies conducted in Iran and elsewhere indicate that burns impose a high burden on families and societies, mainly in developing countries, because of mortality, life-long disability and/or disfigurement.^6^

To combat the high mortality related to burns and the large number of people who suffer from life-long, burn-related disability, burns and their complications can be effectively reduced by applying preventive measures (such as smoke detectors, flame resistant children’s sleepwear) and timely and appropriate treatment methods. Bearing in mind the accidental nature of unintentional burns, prevention is considered to be the most effective method of controlling them.^7,8^

Undoubtedly, accurate statistics on accidents resulting in burn injuries can help to provide effective measures for burns prevention and for the care of injured patients. In Iran, especially in the western region and in Kermanshah province, burns have a relatively high incidence. Furthermore, there have been only a few scattered studies conducted in this field, and those that have been conducted have been of small sample size and suffered from methodological pitfalls.^6,9,10^ In addition, factors affecting injuries such as burns are closely related to the socio-cultural structure of populations and such factors differ among different nationalities and ethnicities. The present study aimed to discover trends in hospital records of burns and investigate the epidemiological characteristics of burns patients in a main referral center in Western Iran. 

## Methods

Kermanshah province is located in Western Iran and borders on Kurdistan province in the North, Iraq in the West, Ilam and Lorestan in the South and Hamedan in the East. It is a mountainous area with a population of around 2 000 000. Imam Khomeini hospital is the main referral center for burns victims not only for Kermanshah province but also for the neighboring provinces. After approval from the ethical committee of Kermanshah University of Medical Sciences, and using a retrospective cross-sectional study, the data on all patients (both admitted and not admitted) who attended the Burns Center at Imam Khomeini Hospital during 2010-2011 and 2011-2012 (24 months) were collected. Age, gender, type of injury (self-immolation or unintentional burns), cause of burns, total body surface area (TBSA), and the time of accident were extracted from the patient files and entered into Excel software. During the two-year study period, only one surgeon was responsible for all burns victims. For the purpose of this study, age was classified in six groups: 0-4, 5-14, 15-24, 25-34, 35-44 and >=45 years old.

Duplicate cases were removed by sorting the name, family name and father’s name. Cases with missing data for the main variables (less than 1%) were removed. Then, according to the TBSA affected by the burns, their severity was divided into three types: mild (<40% of TBSA), moderate (40% to 70%) and severe (>70%). Finally the data were analyzed using the SPSS statistical package (Version 19, for Windows). Mainly focused on descriptive analyses (mean and standard deviation for quantitative variables with normal distribution, median and inter-quartile range for quantitative variables with skewness and frequency for qualitative variables), however, when we compared different groups, we used chi-squared and t-test for two independent samples. Trends in the number of burns were investigated using simple linear regression. For all analysis, a p-value of less than 0.05 was considered statistically significant.

## Results

Overall, 13 248 patients attended the Burns Center at Imam Khomeini in 2010-2011 and 2011-2012 with burn injuries. From the total, 2.5% (326 patients) were admitted to hospital because of self-immolation. The average age of unintentional burn victims was 27±19 years. The median was 26 years (range: 14-41years). The corresponding value for self-immolation cases was 29±13. The number of unintentional burn cases in all age groups, except for the age group 35 years and over, was higher among men (Table 1).While about 20% of unintentional burns occurred in children under 5 years old, the highest proportion of self-immolation cases occurred among 15-34 year-olds (p <0.001).

**Table 1:** Distribution of unintentional burns and self-immolation, Imam Khomeini Hospital, Kermanshah, Iran, March 2010-March 2012

Age group	Unintentional burns				Self-immolaion			
	Male		Female		Male		Female	
	N	%	N	%	N	%	N	%
0-4years	1459	22	1159	18	0	0	0	0
5-14years	648	10	486	8	1	2	14	5
15-24years	1329	20	1398	22	24	45	126	46
25-34years	1291	20	1183	19	20	37	64	23
35-44years	792	12	859	13	6	11	26	10
>=45years	1000	16	1316	21	3	6	42	16
**TOTAL**	**6519**	**100**	**6401**	**100**	**54**	**100**	**272**	**100**

**Figure1:**
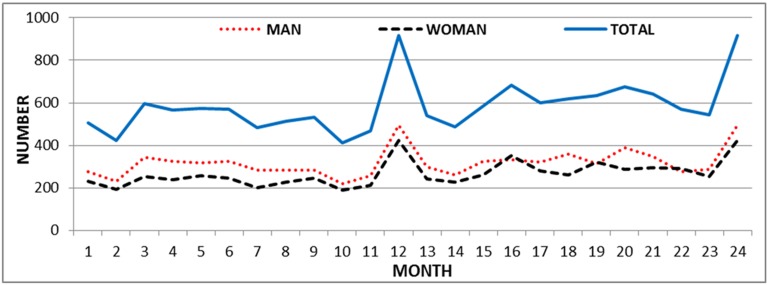
Trends in the number of unintentional burns by gender, Imam Khomeini hospital, Kermanshah, Iran, March 2010- March 2012

In terms of gender, among those with unintentional burns, 6 519 (50.5%) were men while the percentage of men among the self-immolation cases was 16.6% (54 cases) (p <0.001). The number of unintentional burns in various months had a cyclic trend, with the maximum and minimum number of burn cases occurring in March and May respectively (Figure 1). As regards days of the week, the maximum and minimum number of burn cases occurred on Sunday and the weekend days, respectively. But, the observed difference in the number of burn cases on weekdays is not statistically significant (P= 0.31).

The main cause of unintentional burns among the patients referred to the Imam Khomeini Burn Center was hot liquids (6 976 patients (54.0%)) followed by flammable materials (5 168 cases (40.0%)). In the present study, the cause of burns was different among men and women; while hot liquids and flammable materials made up 64.1% and 32.6% of the unintentional burn injuries among women respectively, the corresponding values for men were 47.3% and 44.2% (Table 2). Regarding self-immolation cases, however, all cases involved flammable materials. 

**Table 2:** Distribution of cause of unintentional burns* by gender, Imam Khomeini Hospital, Kermanshah, Iran, March 2010-March 2012

	Flammable materials		hot liquids		hot objects		chemicals		electrical burns	
	N	%	N	%	N	%	N	%	N	%
MAN	3082	59.6%	2880	41.2%	159	64.9%	200	63.9%	198	94.3%
WOMAN	2086	40.4%	4096	58.8%	86	35.1%	113	36.1%	12	5.7%
**Total**	**5168**	**100%**	**6976**	**100%**	**245**	**100%**	**313**	**100%**	**210**	**100%**

*There were no data regarding the cause of burns for 8 cases

**Figure 2:**
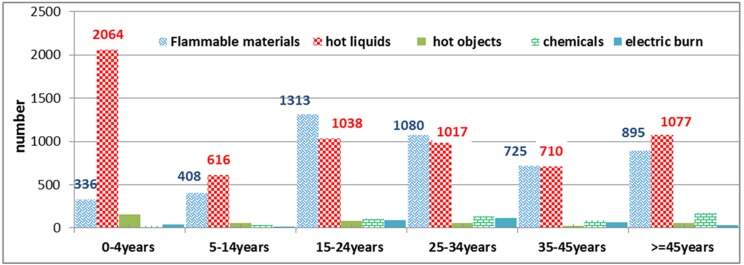
Distribution of cause of burns by age groups, Imam Khomeini Hospital, Kermanshah, Iran, March 2010 – March 2012

Other findings from the present study suggest that the unintentional burn causes were different among different age groups. While hot liquids were the main cause of burns in victims under 5 years and over 45 years old, flammable materials were the main cause of burns in people aged 15 years old and over (P> 0.01) (Figure 2).

With regard to TBSA, the median was 4% (range: 2%-10%). From a total of 12 912 unintentional burn cases with available information about TBSA, 429 (3.3%) were classified as severe mostly caused by flammable materials, 1 027 (8.0%) as moderate and 11 456 (88.7%) as mild (Table 3). However, self-immolations were classified as severe for 305 cases (93.5%). In the present study, 362 cases (85%) of severe burns were caused by flammable materials. In addition, 98% of hot object-induced burns and 99% of chemical-induced burns were mild burns (Table 3).

**Table 3:** Distribution of unintentional burns* by severity, Imam Khomaini Hospital, Kermanshah, Iran, March 2010- March 2012

	Flammable materials		hot liquids		hot objects		chemicals		electrical shock	
	N	%	N	%	N	%	N	%	N	%
Mild	4031	78%	6696	96%	240	98%	303	97%	186	89%
Moderate	775	15%	220	3%	3	1%	7	2%	22	10%
Severe	362	7%	60	1%	2	1%	3	1%	2	1%
**Total**	**5168**	**100%**	**6976**	**100%**	**245**	**100%**	**313**	**100%**	**210**	**100%**

*There were no data regarding the cause of burn for 8 cases

With regards to in-hospital case fatality, while 51% (168 cases) of self-immolation cases died in hospital, the corresponding value for non-intentional burns was 0.33% (43 cases) (p<0.001). Overall the case fatality for 13 246 burn victims was 1.6%.

## Discussion

According to the results of the present study, while about 20% of burns occur in children under 5 years old, this age group constitutes less than 7% of the total population of Kermanshah province according to the Statistical Center of Iran.^11^ The results of the present study suggest that flammable materials and hot liquids are the two most common causes of unintentional burns. This is consistent with the results of studies conducted in various cities in Iran and also the studies conducted in other Western countries.^12-19^ In this study, the average age of subjects was 27 years which is higher than the average age of similar studies conducted in Iran, but, it is lower than the average age of the studies in neighboring countries such as Kuwait and Pakistan.^15,20-25^ The occurrence of burns in lower age groups increases the overall burden of burns compared to other conditions. In addition, although more than 97% of all hospitalized burns cases were related to unintentional cases, most fatalities were related to self-immolations. Such cases were severe in more than 93.5% of cases. Our findings indicate that case fatalities among both self-immolations (51%) and unintentional burns (0.33%) were lower than other reports from elsewhere in Iran.^9,26-28^ Similar to other reports from Iran, the number of self-immolations among women was higher than among men. Lower case fatalities in the present study should be interpreted with caution as some self-immolation cases died before hospital admission and such cases are under-reported in hospital records. In addition, although Imam Khomeini hospital is a referral center, some of the severe cases might have been referred to more advanced centers with a greater number of beds in their intensive care burns units. With regard to the number of self-immolation cases, it is highly likely that the present study suffers from information bias toward under-reporting of such cases as the hospital cost is not paid by health care insurance companies in self-harm cases in Iran.

In terms of the number of burns cases in the various months, as can be seen in Figure 1, burns cases increase dramatically in March due to the last Wednesday of the year (the last Wednesday) when Iranians light bonfires according to an ancient Iranian tradition. This increase is consistent with similar studies. ^21,23^ In May, the number of burns cases is lower than in other months. Although the trend and the effect of the season were determined, it seems that it is essential to study the effect of months on the number of burns cases over a longer time period such as 5 years. 

Other findings of this study suggest that the number of burn cases in men was higher than in women, which is consistent with the results of studies in Esfahan and Urmia and also studies in Taiwan, Africa, U.K and USA. ^8,28-33^ But it is not similar to the results of studies conducted in Shiraz, Kurdistan, Pakistan and Turkey. ^10,17,22,25^ Although social and cultural factors may affect such differences in studies, a different age structure of samples in various reports may contribute to the observed differences. As can be seen in Figure 2 , different factors can cause burns in different age groups. In the under-5 age group, hot liquids were the main cause of burns, which is fully consistent with the results of a study that examined burns in children and adolescents under 15 years old in Isfahan and also studies that were conducted in Western countries. ^8,34,35^ In the case of adolescents, flammable materials were the main cause of burns. This finding is also consistent with similar studies. ^6,36,37^ The main cause of burns was also hot liquids among people aged over 45 years. The present work is a cross-sectional study using hospital data and therefore suffers from certain limitations. There is still a need to investigate the real burden of the problem within population-based studies and unfortunately there is no accurate figure on the incidence of the problem as well as related risk factors. However, this study presents an updated figure from burns referred to Imam Khomeini Hospital, the main referral center for burns in Western Iran. In addition, the present study is one of the largest cross-sectional studies using hospital records from Western Iran, where there is paucity of information. In fact, burns are a major public health issue, especially in developing countries, that can be prevented most effectively by applying preventive measures. In the present study similar to other reports from Iran, flammable materials plus hot liquids were the major causes of unintentional burns. While application of strategies such as smoke detectors and hot water temperature regulations in low- and middle-income countries such as Iran are inadequate, increasing public awareness using mass media may address the issue among such countries to some extent. With regards to self-immolation, using mass media with more emphasis on cultural as well as religious issues can decrease suicide attempts and fatalities. Future epidemiologic studies may focus on the real size and risk factors of burns within a population-based study.
